# Heat tolerance of goats to increased daily maximum temperature and low salinity of drinking water in tropical humid regions

**DOI:** 10.5713/ab.23.0288

**Published:** 2024-01-20

**Authors:** Asep Indra Munawar Ali, Sofia Sandi, Lili Warly, Armina Fariani, Anggriawan Naidilah Tetra Pratama, Abdullah Darussalam

**Affiliations:** 1 Faculty of Agriculture, Universitas Sriwijaya, South Sumatra, 30662, Indonesia; 2 National Research and Innovation Agency, Jakarta, 10340, Indonesia

**Keywords:** Behavior, Goat, Heat Stress, Maximum Temperature, Saline Drinking Water

## Abstract

**Objective:**

The daily maximum temperature and seawater level continuously increase as global warming continues. We examined the adaptability and production performance of heat-stressed goats with a supply of low-saline drinking water.

**Methods:**

Twelve Kacang and Kacang Etawah cross goats were exposed to two climatic conditions (control, 25°C to 33°C, 83% relative humidity [RH], temperature humidity index [THI]: 76 to 86; and hot environment, 26°C to 39°C, 81% RH, THI: 77 to 94) and two salt levels in drinking water (0% and 0.4% NaCl). The experimental design was a Latin Square (4×4) with four treatments and four periods (28 days each).

**Results:**

Temperature of the rectal, skin, and udder, and respiration rate rose, reached a maximum level on the first day of heat exposures, and then recovered. Plasma sodium rose at 0.4% NaCl level, while the hot environment and salinity treatments increased the drinking water to dry matter (DM) intake ratio. Water excretion was elevated in the hot environment but lowered by the increase in salinity. Total lying time increased, whereas change position frequency decreased in the hot condition. Lying and ruminating and total ruminating time increased and explained the enhanced DM digestibility in the hot conditions.

**Conclusion:**

The goats exhibited a high level of plasma sodium as salinity increased, and they demonstrated physiological and behavioral alterations while maintaining their production performances under increasing daily maximum temperatures.

## INTRODUCTION

Sea level rise is a crucial issue that affects vital agricultural sectors and jeopardizes access to clean water supply as global warming progresses. The rise in sea level is primarily caused by seawater expansion and meltwater from mountain glaciers and polar ice sheets. Since 1880, the global average seawater level has risen about 21 to 24 cm, with an annual rise of 3 mm. The Intergovernmental Panel on Climate Change projected that global seawater level will rise from 0.43 to 0.84 m by 2100, depending on the underlying scenario [1,2]. As a result, seawater intrusion into groundwater and increased floods will pose emerging threats to the drinking water supply for livestock production in coastal regions [3]. Moreover, under climate change, the global surface temperature is rising and expected to continue in the future [4]. The most direct consequences of global temperature change in tropical regions are higher daily mean and maximum temperatures [5–8].

Goat farming is recognized as one of the farming sector’s backbones, particularly for small-scale farmers, due to its low investment and managerial skills, and the goats’ high fertility rate, and adaptability [9]. Concerning the potential contamination of drinking water by seawater salinity, the detrimental effect of high-saline drinking water on livestock physiology and performance has been extensively investigated [10–13]. Sodium chloride salt is an essential nutrient in animal diets that plays a role in the body fluid balance, nerve function, thermoregulation responses, and the absorption of water and glucose. Excess salt, on the other hand, causes impaired cellular osmosis and hypertension and could lead to cardiovascular and renal dysfunction and lowered production [14]. Under extreme daily maximum temperatures, drinking water intake maintains water and electrolyte balance due to evaporative body water loss, where blood plasma electrolytes (Na^+^ and K^+^) decreased during heat stress [15,16]. For instance, adding 0.4% NaCl to drinking water reduced body temperature and improved body weight (BW) and feed conversion ratio [17]. The 0.4% NaCl addition also positively affected growth and sexual maturity in African Dwarf kids [18]. Therefore, low-saline drinking water could help maintain the electrolyte balance in heat-stressed goats.

The thermal environment influences animal physiology. For instance, in a coastal region with tropical humid climate, goats experience heat stress when the daily temperature humidity index (THI) exceeds 86 (daily maximum temperature 34°C at 70% relative humidity [RH]) in the afternoon [19,20]. When THI rises, they respond physiologically and behaviorally to maintain core body temperature. Increased body parts temperature, respiration rate, drinking water intake, and lowered feed intake are most important physiological symptoms of heat stress [21]. Seeking shade, changing in lying and standing durations, and decreased body activity were described as behavioral responses [16], which might be considered the primary sign of comfort under heat-stress conditions. However, the literature on the effect of the higher daily maximum temperature on tropical goat breeds that experienced daily heat stress is limited. The information could be valuable in determining future adaptation priorities and options for the livestock sub-sector resilience and sustainable production. Therefore, this study determined the impacts of the higher daily maximum temperature and low salinity of drinking water on the physiological and behavioral responses of tropical goats.

## MATERIALS AND METHODS

### Study animals, feeding management, and treatments

This experiment was approved by the animal care and use committee of the Faculty of Agriculture, Universitas Sriwijaya (No. KPPHP-2022–4). Twelve female Kacang and Kacang Etawa Crossbreeds (BW, 14.0±0.7 and 18.4±0.9 kg, respectively) between ten to twelve months old were used in a 112-day trial from November 2022 to February 2023. During the trial, the goats were weighted weekly and maintained in individual metabolic pens (1.5×0.75 m) designed to ease the collection of fecal and urine excretion and feed samples.

The goats were offered chopped Guinea hay and a concentrate mixture (57.5% corn meal, 23.3% copra meal, 15% fish meal, 3% molasses, 0.3% urea, and 1% mineral mix, dry matter [DM] basis). The animals were fed (*ad libitum*) with hay twice a day at 09:00 and 16:00 in equal portions, whereas the concentrate was offered at 2.5% of BW at 09:00.

The experiment was conducted as a complete Latin Square (4×4) with four treatments, four periods, and each lasting 28 days. The goats were divided into three groups based on their initial BW and each goat was allocated into the four treatment groups of three goats each. The groups were switched to the other treatments in the next periods and the treatments order was balanced to minimize carryover effects [22]. The treatments were combinations of two climatic conditions (control, 25°C to 33°C, 83% RH, THI: 76 to 86; and hot environment, 26°C to 39°C, 81% RH, THI: 77 to 94) and two salinity levels (0% and 0.4% NaCl). The THI values were followed formula of Thom [23] as follows: THI = 0.8×T+((RH/100)×(T–14.3))+46.4 (Thom [23]), where T is air temperature and RH is the relative humidity.

The experiment was conducted in two climatic rooms. The climatic environment in the control treatment room followed the daily climatic environment outdoors, where in the hot treatment room, the daily maximum temperature was increased to ≤39.2°C by electric air heaters equipped with a thermostat (XH W3001) from 10:00 to 16:00. The climatic data during the four experimental treatments is presented in Table 1. Drinking water was provided *ad libitum* throughout the experimental periods. The 0.4% saline water was prepared by adding a defined amount of 99% pure salt (Braun Ltd., Mumbai, India) to the well water. The characteristics of the drinking water offered to the goats are shown in Table 2.

### Measurements, sample collection, and analysis

Data on indoor ambient temperature and RH in both experimental rooms were recorded continuously every 10 minutes throughout the study using two data loggers (Benetech G1365; Shenzhen Jumaoyuan Science & Technology Co., Ltd, Shenzhen, China). The drinking water sample was collected each week, and then the samples were pooled at the end of each period. The water samples were analyzed to determine pH, electrical conductivity (Hanna HI 98130; Hanna Instruments, Inc., Nusfalau, Romania), total dissolved solids (Milwaukee Mi 180 Bench Meter; Milwaukee Instruments, Inc., Rocky Mount, NC, USA), sodium, and chlorides (inductively coupled plasma atomic emission spectroscopy Varian 715-ES; Agilent, Santa Clara, CA, USA).

For thermo-physiological parameters, rectal (RT), skin (ST), udder temperatures (UT), and respiration rates (RR) were recorded at 0 (11:00), 3, 6, 9, 24, 48, 72, and 240 hours after transferring the goats to the hot conditions. The RT was measured using a digital clinical temperature (Omron MC-343F; Omron Healthcare Singapore Pte Ltd, Singapore), while the UT and ST (in a shaved area at the shoulder) were measured with an infrared thermometer (Beureur FT 90; Beurer Indonesia, Jakarta, Indonesia) at ±2 cm. The RR was determined by counting the flank movements when the goat was lying.

On day 12 of each period, a blood sample of each goat was obtained from the vena jugularis between 11:00 and 12:00. The samples were transferred to an ethylenediaminetetraacetic acid-containing blood tube and then placed in box ice. Other blood samples were also taken in a blood tube containing a gel clot activator for glucose, urea, and creatinine analysis, as well as a blood tube containing heparin for the determination of blood plasma electrolytes. The samples were then transported to the laboratory for hematological analysis. Hemoglobin, erythrocytes, leukocytes, hematocrit, mean corpuscular volume (MCV), mean corpuscular hemoglobin (MCH), and mean corpuscular hemoglobin concentration (MCHC) levels were measured with Sysmex XP-100. Glucose, cholesterol, and creatinine concentrations were measured with Humastar 100 (Human Diagnostic Asia Pacific Pte Ltd, Singapore), while blood plasma electrolytes (Na^+^, K^+^, Cl^−^) were measured with Amtast CBS-400 (Amtast USA Inc., Lakeland, FL, USA).

Throughout the trial, feed and water consumption were recorded daily. Samples of offered and refused feed and fecal excretion were collected over six consecutive days of the collection week (days 22 to 28) to determine feed intake and digestibility. The hay and concentrate (±100 g each) were sampled twice a week, while refusal feed (±100 g) and feces (±10% of the daily fresh defecation) were sampled daily. The composted samples were dried at 45°C for 72 hours and then ground through a 1-mm mesh for DM content analysis.

The determination of the water balance was also established during the collection week. For the estimation of daily water evaporation, two identical buckets were placed in each room. The buckets were weighed each morning, and the values were used to correct the water intake. Meanwhile, urinary water excretion was determined by weighing the daily urine excretion. A 40-mL of urine sample was taken and stored (−20°C), and at the end of each period, the samples were pooled per animal, and 3 mL samples were then dried at 60°C for two days to correct the water content of the urine. Water retention was calculated from the difference between total water intake and excretion in the urine and feces.

The behavioral responses of goats were observed three times per period at weeks 2 and 3 using a camera system (Hikvision, Hangzhou Hikvision Digital Technology Co., Ltd., Zhejiang, China) for six hours from 10:00 to 16:00. The goat activities such as standing idle, standing, and ruminating, lying idle, lying, and ruminating, and eating were quantified at five-minute intervals to determine the time spent on the behavioral responses. Moreover, the change in position (lying to standing and reverse) and the frequency of urination and defecation were counted during the six hours of recording.

### Statistical analysis

The data generated from four treatments, four periods, and twelve goats was analyzed using the PROC MIXED procedure of the statistical analysis system (SAS Inst., Inc., Cary, NC, USA). The treatments (thermal environment and salinity), experimental periods, and their interactions were included in the mixed model as fixed effects and the animal as a random effect. For the behavioral data, the repeated measurements were accounted for via sampling day with compound symmetry as a covariance structure. The significance level was set at p<0.05 and the results are presented as arithmetic means ±standard error.

## RESULTS

There were significant increases in the thermo-physiological response of goats during hot temperatures compared to the control ambient temperature (p<0.01). The body parts temperature rose by 0.2°C to 0.8°C, while RR increased 36 times per minute in the hot thermal environment. The sodium ion concentration was higher at 0.4% compared to 0% salinity (p<0.05); however, there was no significant effect of the treatments on the other hematological parameters (Table 3).

Feed and water intake were not influenced by the salinity levels or thermal environment (p>0.05), but significantly varied during the experimental periods (p<0.01). The DM digestibility was improved by 7.7% (p<0.01) by the thermal environment, whereas the ratio of water and feed intake was increased by the hot environment and 0.4% salinity (p<0.05). Further, water excretions (g/kg BW^0.75^) from urine and feces were raised by 0.4% salinity (p<0.01), resulting in an increase in the amount of excreted water per intake of water (p<0.01). On the other hand, the water excretion per consumed water was lowered by the higher daily maximum THI (p<0.05). The water excretion was elevated by 7.6% at the 0.4% salinity but decreased by 3.9% at the hot ambient temperature. Subsequently, the daily gain of the goats was not influenced by the treatments (p>0.05) (Table 4). Moreover, the experimental periods affected water intake and excretion (p<0.01), but there was no significant effect of interactions on the parameters (data not shown).

There was no significant influence of the salinity level on the behavioral responses of the goats (p>0.05), while the time for eating hay, total eating time, standing idle, total standing, and total lying time, as well as the frequency of changing positions, urinating, and defecating, varied during the experimental periods (p<0.05). Drinking time was longer at the higher THI level compared to the control thermal environment (p<0.01). Further, standing idle and total standing duration were reduced at the higher THI level. Lying and ruminating, and total lying time were longer in the higher THI (p<0.05). Changes in position and frequency of urination and defecation were decreased (p<0.01) in the higher THI level (Table 5).

## DISCUSSION

### Thermo-physiological and hematological responses

**Figure 1:**
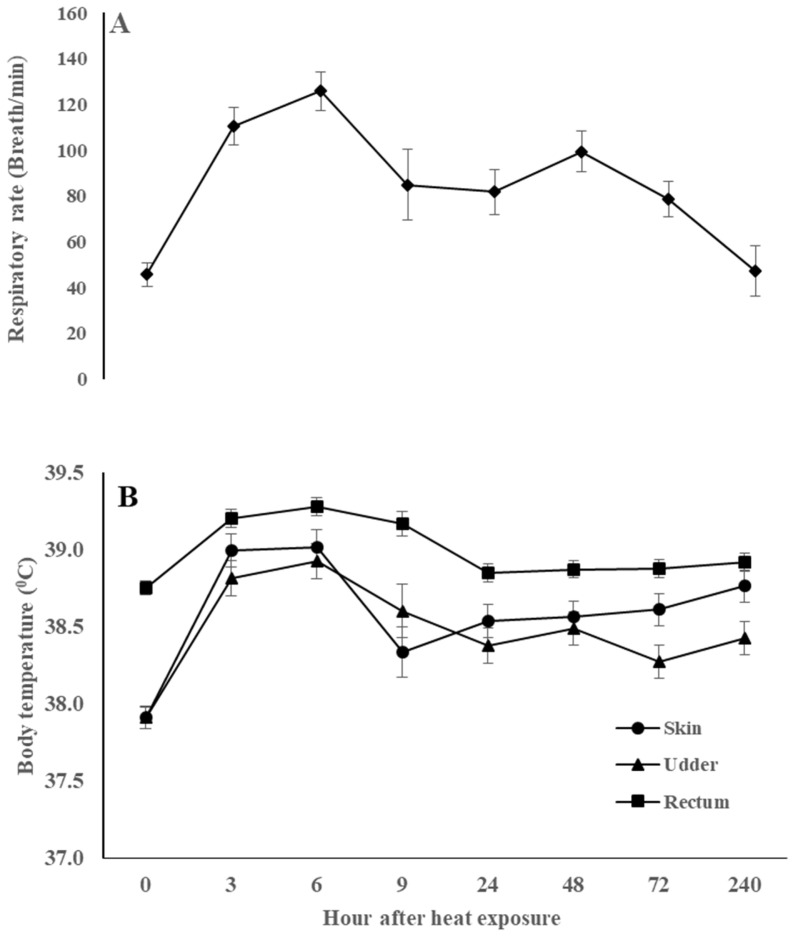
Change of respiration rate (A) and temperature of skin, udder, and rectum (B) of goats exposed to a hot thermal environment. The standard errors are shown as vertical bars. The thermoregulation responses reached a maximum level on the first day of heat exposure.

In tropical humid regions, optimal livestock production is partially limited by the high daily maximum temperature and RH, and the risk of severe heat stress is projected to be elevated in many tropical regions as global warming progresses [19]. With current climatic conditions, the goats in the present study experienced high levels of heat stress for more than 12 hours per day when THI levels were ≥79 Serradilla et al [20]. As homeotherms, the elevated THI levels in the hot thermal environment treatment (≤94) impacted the thermo-physiological systems to maintain their normal body temperature by increasing the body temperature and the evaporative respiratory heat disposal mechanism [16]. The elevated level of thermoregulation response was seen three and six hours after heat exposure (p<0.05). However, after 24 hours, the body parts temperature and breath rate were lowered, indicating a partial adaptation to the heat stress conditions (Figure 1). A similar finding was also reported by previous studies on goats suffered maximum heat stress during the first [24] to third days [25,26] of heat exposure.

The RT range in this study (38.1°C to 40.0°C) was comparable to that of Brazilian breed goats (38.3°C to 39.5°C), while the ST range (36.0°C to 40.2°C) was higher than that of those breeds (31.4°C to 38.6°C) [21]. A similar range of ST (35.5°C to 40.5°C) was reported in a tropical semi-arid region when the daily maximum temperature reached 43.5°C [27]. However, the RR range in the previous study (27.5 to 38.0 breaths/min) was much lower than the present work (14.9 to 183.1 breaths/min). Similar results (38.3°C to 40.4°C and 30 to 150 breaths/min, for RT and RR, respectively) were reported in Murciano-Granadina goats [25]. The difference was probably due to variances in the treatment conditions, breed, and physiological states.

As shown in Table 3, hematological responses were not significantly different between the treatments. Plasma potassium and chloride levels did not change, while sodium levels were higher at the 0.4% than at 0% NaCl but still within the ranges in previous studies [10,12,28]. Compared to the previous study, elevated sodium concentrations were also reported in goats with higher levels of salinity in drinking water [10,28]. Thiet et al [12] reported that the sodium, potassium, and chloride concentrations did not differ between two levels of salinity, while Zoidis and Hadjigeorgiou [28] and Runa et al [10] found an increase in the electrolyte levels. The urea and creatinine levels represent renal function; an increase in salt concentration and extended exposure may result in a rise in the levels [10,28].

In this study, the increase in RR did not result in a higher hemoglobin level. The elevated RR was linked to an increase in oxygen intake [16]. Abdelatif et al [29] also reported the non-significant influence of THI levels on the erythrocytes, leukocytes, MCV, and MCHC levels in Nubian goats. However, Alam et al [30] found higher hemoglobin, erythrocytes, and leukocytes levels in heat-stressed goats. Considering the effect of the salinity levels, Tulu et al [13] also reported that the levels of hemoglobin, erythrocytes, leukocytes, MCV, and MCHC were not changed.

When feed intake was reduced, a lower glucose level was associated with higher insulin activity [16,21,31–33], whereas Hamzaoui et al [25] found a non-significant change in glucose levels when food intake decreased in lactating goats. The inconclusive results were also observed in cholesterol levels when the animals experienced heat stress. The increased cholesterol level was associated with triglyceride mobilization [21,34], while the decrease was related to increased water retention, lowered thyroid activity, and feed (cholesterol) intake [16,31,33,35]. In the current study, it appears that feed intake had a greater impact on glucose and cholesterol levels than the major shift in body water retention. This also applied to the effect of the salinity levels; the insignificant change in glucose levels in the present study seems to be related to the intake level [28].

### Feed intake and water balance

The decreased feed intake and increased water intake in the heat-stressed animals are interpreted as an attempt to minimize heat production and maintain body fluid balance [16,21]. In the present study, the feed and water intake were not changed, but the ratio of water to feed intake was increased by THI and salinity levels. Since the measurement was conducted on days 22 to 28, according to the thermo-physiological responses (Figure 1), the non-significant change in feed intake may represent a goat’s capacity to adjust to the increased THI level. Moreover, the elevated drink water intake (p = 0.0517) at the 0.4% NaCl level might be related to the significant increase in plasma sodium concentration (Table 3) and urinary and fecal water excretion (Table 4) to maintain sodium balance by increasing water intake and excretion. Similar results were also reported in deer [36], lamb [37], and goat [28]. The increased THI, on the other hand, reduced water excretion in order to maintain body water retention (Table 4) for the evaporative cooling process [25]. It appears that a higher level of salinity (≥0.4%) could have adverse effects on heat-stressed goats.

### Behavioral responses

The duration and frequency of the various activities reflect goat comfort under hot conditions. Table 5 shows the observed behavioral indices in goats, where only the thermal environment treatment altered the behavioral responses. The increased drinking duration in hot conditions corresponded to the significant increase in the drinking water/feed intake ratio (Table 4) to maintain the body fluid balance.

Furthermore, total lying was longer than standing time in both thermal conditions, and the goats spent more time lying at the elevated THI level. The longer time spent lying during the hot conditions might be an adaptive behavior to reduce additional heat production from body movements, and this corresponded to the decrease in change position frequency. The longer lying time during heat stress was also reported in goats [26,38,39] and sheep [40], but another study in goats [41] found that lying time was lowered during heat stress. In addition, Salama et al [26] reported that changing the position increased under heat stress, reflecting goats’ uncomfortable behavior. The opposing reaction may be related to the period of behavioral observation. The observation in their study was conducted under maximal heat stress (day 3), whereas the observation in this study was taken after the first week of each experimental period.

The longer total ruminating time in the hot conditions could be related to the significant improvement of DM digestibility (Table 4). Chewing time increased as ruminating time increased, and was attributed to a lower feed particle size and a higher ruminal fermentation efficiency [42,43]. A higher digestibility as a result of the lowered feed intake was reported in goats [44] and heifers [45] during hot environments. However, an opposite result was evidenced in cows [46] and goats [38] where ruminating time was shorted during heat stress conditions. In the present study, the goats spent more time for rumination in the lying position than in the standing position. Previous studies reported that rumination time was related to the lying time in dairy cows, indicating that periods of rumination were more frequent when the animal were lying down [47,48].

### Experimental periods effect

The significant experimental period effects were found for several parameters. Climatic conditions during the four periods (Table 1) fluctuated and significantly influenced the animal responses (p<0.05). The daily maximum THI elevated for only a six-hour period (10:00 to 16:00), and although significant changes in the animal responses were observed, the influence of the fluctuation of temperature and RH during the non-treatment period (16:00 to 10:00) could not be neglected.

## CONCLUSION

The study’s findings suggest that goats exposed to 0.4% NaCl saline water had greater plasma sodium levels, water intake, and excretion but no beneficial effect on physiological and behavioral responses during hot conditions. The higher maximum THI levels increased water intake and retention and affected the thermo-physiological and behavioral response of goats, while longer ruminating time resulted in a higher DM digestibility. The present study demonstrated the goat’s capabilities to cope with an increasing daily maximum temperature, which was also influenced by the duration of the maximum temperature and diurnal climatic conditions.

## Figures and Tables

**Table 1:** Climatic data collected in control and high daily maximum temperature humidity index (hot) during the four experimental periods

Parameters	Experimental periods			

	1	2	3	4
Control				
Maximum temperature (°C)	33.0	32.1	32.4	32.5
Minimum temperature (°C)	25.4	25.2	25.0	25.4
Mean temperature (°C)	28.5	27.9	28.0	28.1
Relative air humidity (%)	82.5	84.0	82.0	81.9
Maximum THI^1)^	86.2	85.5	85.6	85.6
Minimum THI	76.6	76.1	75.5	76.2
Hot				
Maximum temperature (°C)	38.7	39.2	38.1	39.0
Minimum temperature (°C)	26.0	26.1	25.8	26.1
Mean temperature (°C)	30.4	30.4	30.2	30.1
Relative air humidity (%)	80.2	80.4	82.4	82.7
Maximum THI	92.9	93.8	93.1	93.8
Minimum THI	77.4	77.7	76.3	77.2

1) Temperature humidity index (THI) = 0.8×T + ((RH/100) ×(T − 14.3)) +46.4 (Thom [23]), where T is air temperature and RH is the relative humidity.

**Table 2:** Concentrations of substances in drinking water offered to treatment groups

Parameters	NaCl	

	0%	0.4%
Sodium (mg/L)	7.9	754.5
Chlorides (mg/L)	139.9	1,243.5
Total dissolved solids (mg/L)	42.3	377.7
Electrical conductivity (mS/cm)	0.1	7.6
pH	5.3	6.2

**Table 3:** Thermo-physiological and hematological responses of goats (mean±standard error) in control and hot thermal environment offered water having two different salinity levels^1)^

Parameters	Control		Hot		Effects (p-values)		
		
	0% NaCl	0.4% NaCl	0% NaCl	0.4% NaCl	Period	Thermal environment	Salinity
Skin temperature (°C)	38.0±0.10	37.9±0.11	38.7±0.05	38.8±0.07	<0.0001	<0.0001	0.2718
Rectal temperature (°C)	38.8±0.05	38.7±0.04	39.0±0.03	39.0±0.04	<0.0001	<0.0001	0.8782
Udder temperature (°C)	37.9±0.09	37.9±0.11	38.6±0.06	38.5±0.07	<0.0001	<0.0001	0.4262
Respiratory rate (breath/min)	54.6±5.31	51.4±5.63	85.9±5.89	92.6±6.05	0.0042	<0.0001	0.8725
Hemoglobin (g/dL)	11.0±0.25	11.0±0.29	11.1±0.31	10.7±0.29	0.0099	0.5047	0.2520
Erythrocytes (10^6^/μL)	4.5±0.23	4.6±0.34	4.5±0.36	4.3±0.24	<0.0001	0.3623	0.7377
Leucocytes (10^3^/μL)	16.1±1.06	16.9±1.27	16.5±1.06	15.6±0.92	0.3986	0.5653	0.9184
Hematocrit (%)	46.1±4.41	48.4±3.49	47.9±3.69	47.1±3.05	0.0046	0.9163	0.7459
MCV (fL)	110.5±1.77	105.5±2.88	106.7±2.59	108.3±2.54	0.1136	0.8149	0.4462
MCH (pg)	25.0±0.98	25.2±1.61	25.9±1.63	25.4±1.26	<0.0001	0.1511	0.6939
Creatinin (mg/dL)	0.3±0.11	0.3±0.08	0.3±0.08	0.3±0.10	<0.0001	0.5980	0.3748
Glucose (mg/dL)	70.3±1.60	68.3±1.60	67.4±1.28	68.2±2.20	0.0136	0.2182	0.6142
Urea (mg/dL)	19.5±2.20	19.3±2.18	20.6±2.11	19.0±1.70	<0.0001	0.7506	0.5106
Cholestrol (mg/dL)	138.3±10.06	135.0±5.46	131.7±6.90	137.8±9.13	0.3020	0.7924	0.8457
Sodium (mmol/L)	140.1±0.56	141.7±0.93	139.9±0.62	141.6±0.84	0.9553	0.8659	0.0362
Cloride (mmol/L)	111.1±0.96	111.0±0.66	110.2±1.03	111.6±0.98	0.2454	0.8493	0.4498
Potasium (mmol/L)	4.5±0.09	4.5±0.06	4.6±0.08	4.5±0.09	0.1253	0.6377	0.6377

MCV, mean corpuscular volume; MCH, mean corpuscular hemoglobin; MCHC, mean corpuscular hemoglobin concentration.

1) Range of daily temperature humidity index: 76 to 86 and 77 to 94 for control and hot thermal environment, respectively.

**Table 4:** Dry matter intake, digestibility, body water balance, and gain of goats (mean±standard error) in control and hot thermal environment offered water having two different salinity levels^1)^

Parameters	Control		Hot		Effects (p-values)		
		
	0% NaCl	0.4% NaCl	0% NaCl	0.4% NaCl	Period	Thermal environment	Salinity
DM intake (g/kg BW^0.75^)							
Hay	8.1±1.02	6.7±0.60	7.0±0.71	8.1±0.81	0.2301	0.8473	0.8167
Concentrate	38.2±2.18	39.2±2.26	38.6±2.52	38.3±2.79	0.0345	0.8523	0.8109
Total	46.3±1.82	45.9±1.95	45.6±2.37	46.4±2.43	0.0016	0.9089	0.8763
DM digestibility (%)	49.7±3.13	47.0±4.21	56.2±3.72	56.0±3.76	<0.0001	0.0012	0.4940
Water intake (g/kg BW^0.75^)							
Drinking (DW)	78.7±6.03	88.8±8.32	87.0±7.84	94.0±6.81	<0.0001	0.1182	0.0517
Feeding	3.2±0.31	3.0±0.32	3.1±0.38	3.2±0.41	<0.0001	0.7534	0.8861
Total	81.8±6.20	91.8±8.51	90.1±8.08	97.2±7.09	<0.0001	0.1236	0.0573
DW/DM intake	1.69±0.108	1.93±0.154	1.90±0.126	2.03±0.106	0.0010	0.0406	0.0148
Water excretion (g/kg BW^0.75^)							
Urine	20.7±3.69	31.8±5.23	22.4±4.68	28.1±3.56	0.0025	0.7270	0.0064
Feces	11.4±0.74	12.9±0.89	11.5±0.90	13.0±0.99	0.0128	0.8709	0.0219
otal	32.1±4.21	44.7±5.31	33.9±5.13	41.2±4.26	0.0006	0.7650	0.0024
Water retention (g/kg BW0.75)	49.7±3.13	47.0±4.21	56.2±3.72	56.0±3.76	<0.0001	0.0012	0.4940
Water excretion/intake (%)	37.9±2.72	47.6±2.71	36.1±2.21	41.6±1.96	0.1375	0.0322	0.0002
Daily gain (g)	366.2±35.74	325.7±47.04	318.3±66.82	291.1±58.26	0.0369	0.2856	0.3787

DM, dry matter; BW, body weight.

1) Range of daily temperature humidity index: 76 to 86 and 77 to 94 for control and hot thermal environment, respectively.

**Table 5:** Behavioral responses of goats (mean ± standard error) in control and hot thermal environment offered water having two different salinity levels^1)^

Parameters	Control		Hot		Effects (p-values)		
		
	0% NaCl	0.4% NaCl	0% NaCl	0.4% NaCl	Period	Thermal environment	Salinity
Eating time (min)							
Hay	27.5±4.37	29.9±2.79	30.8±2.85	31.4±3.15	<0.0001	0.9660	0.4108
Concentrate	36.8±2.80	38.9±3.52	36.5±3.14	34.6±3.06	0.1038	0.5223	0.7904
Total eating time	64.3±4.91	68.8±4.35	67.4±4.88	66.0±4.34	0.0250	0.6565	0.4770
Drinking time (min)	1.5±0.48	0.8±0.31	2.2±0.54	2.9±0.78	0.5912	0.0241	0.5689
Standing time (min)							
Standing idle	54.4±3.28	48.9±3.94	37.2±2.77	41.0±3.51	0.0165	<0.0001	0.4956
Standing and ruminating	1.8±0.45	2.8±0.83	3.8±0.88	2.9±0.81	0.9141	0.0531	0.9451
Total standing time	56.3±3.43	51.7±4.30	41.0±2.79	43.9±3.82	0.0232	0.0005	0.5371
Lying time (min)							
Lying idle	193.5±6.41	204.7±5.68	193.1±6.66	193.3±7.34	0.0541	0.4304	0.0567
Lying and ruminating	59.2±3.64	49.0±3.18	71.4±3.48	68.5±3.88	0.2548	<0.0001	0.0983
Total lying time	252.6±6.45	253.8±6.01	264.4±6.17	261.8±5.78	0.0007	0.0162	0.2984
Total Ruminating (min)	61.0±3.55	51.8±2.99	75.1±3.18	71.4±3.85	0.2911	<0.0001	0.0932
Change in position frequency^2)^	18.4±1.33	20.8±1.68	18.6±1.05	15.4±0.87	0.0007	0.0472	0.5416
Urination frequency	1.4±0.14	1.8±0.23	1.2±0.17	1.2±0.14	<0.0001	0.0061	0.4424
Defecation frequency	3.2±0.21	3.4±0.27	2.6±0.24	2.9±0.27	<0.0001	0.0071	0.5707

1) Range of daily temperature humidity index: 76 to 86 and 77 to 94 for control and hot thermal environment, respectively; behavioral measurement from 10:00 to 16:00.

2) Lying to standing and vice versa.

## Data Availability

The datasets analyzed during the current study will be available in repository of author’s institution.
